# Cost-effectiveness analysis of cadonilimab plus chemotherapy as a first-line treatment option in HER-2-negative advanced gastric cancer

**DOI:** 10.3389/fpubh.2025.1644176

**Published:** 2025-07-21

**Authors:** Longfeng Zhang, Qingsheng Yang, Zhiwei Zheng

**Affiliations:** ^1^Department of Thoracic Oncology, Clinical Oncology School of Fujian Medical University, Fujian Cancer Hospital, Fuzhou, Fujian, China; ^2^Department of Acupuncture and Rehabilitation, Shantou Hospital of Traditional Chinese Medicine, Shantou, China; ^3^Department of Pharmacy, Cancer Hospital of Shantou University Medical College, Shantou, China

**Keywords:** advanced gastric cancer, cadonilimab, chemotherapy, cost-effectiveness analysis, HER-2-negative

## Abstract

**Objective:**

This study aims to evaluate the cost-effectiveness of using cadonilimab plus chemotherapy compared to chemotherapy in HER-2-negative advanced gastric cancer from the perspective of the Chinese healthcare system.

**Methods:**

A cost-effectiveness analysis was conducted utilizing a partitioned survival model to simulate the expected costs and outcomes associated with the treatment of patients with cadonilimab in combination with chemotherapy versus chemotherapy over a 10 years lifetime horizon. Cost data were sourced from published literature and national databases. Data on treatment efficacy, adverse events, and transition probabilities were derived from the phase 3 COMPASSION-15 trial. The WTP threshold in this study was established at 40,343.68 USD per QALY. Sensitivity analyses were performed to evaluate the robustness of the results and assess the impact of variations in key parameters on the cost-effectiveness outcomes.

**Results:**

The base case analysis revealed that in all population of randomized patients, treatment with cadonilimab resulted in an incremental gain of 1.08 QALYs compared to chemotherapy, at an incremental cost of 58,862.61 USD. The ICER for this cohort was calculated to be 54,502.42 USD per QALY. In the subgroup of patients with a PD-L1 CPS ≥ 5, patients treated with cadonilimab experienced a greater increase in 1.33 QALYs compared to chemotherapy, at an incremental cost of 35,661.87 USD. The ICER for this subgroup was 26,813.44 USD per QALY. Sensitivity analyses conducted demonstrated the robustness of the results to variations in model inputs and assumptions. Moreover, the probabilistic sensitivity analysis indicated that cadonilimab in combination with chemotherapy had a 4.70 and 93.90% probabilities of being considered cost-effective at a WTP threshold of 40,343.68 USD per QALY for the all randomized patient group and the subgroup of patients with a PD-L1 CPS ≥ 5, respectively.

**Conclusion:**

The addition of cadonilimab to standard chemotherapy for first line treatment of HER-2-negative advanced gastric cancer may not be considered a cost-effective option compared to chemotherapy alone. However, for the subgroup of patients with PD-L1CPS ≥ 5, the ICER was 26,813.44 USD per QALY, indicating that this treatment approach could potentially be deemed cost-effective in China.

## 1 Introduction

Gastric cancer is the fifth most common cancer worldwide in terms of both incidence and mortality, with nearly 1 million new cases diagnosed annually and resulting in over 650,000 deaths globally (1). The incidence of gastric cancer demonstrates significant geographical variation, with the highest rates observed in Asia (2). China accounts for a significant proportion of global incident cases, with approximately 43.9% of cases attributed to the country (3). Gastric cancer has become a significant public health concern in China, ranking as the fifth most common cancer and the third leading cause of cancer-related deaths in China (4). It is estimated that there will be approximately 359,000 new cases of gastric cancer, leading to 261,000 deaths in China. This accounts for approximately 10.14% of all cancer-related fatalities in China (5). The rising incidence and mortality rates of gastric cancer underscore the urgent need for effective prevention, early detection, and treatment strategies to address this growing health challenge. Despite advancements in diagnostic and therapeutic modalities, more than half of patients with gastric cancer are diagnosed with metastatic disease that is not amenable to surgical intervention (6). The most common histological subtype of gastric cancer is adenocarcinoma, with a particular emphasis on the human epidermal growth factor receptor 2 (HER-2) negative subtype (7, 8). Advanced gastric cancer, especially in HER-2-negative patients, represents a pressing clinical dilemma characterized by its high aggressiveness and limited therapeutic alternatives (9). In recent years, the emergence of immunotherapeutic agents, particularly immune checkpoint inhibitors (ICIs), has revolutionized the treatment landscape for HER-2-negative advanced gastric cancer (10, 11). The use of ICIs in advanced gastric cancer represents a promising strategy to overcome the limitations of conventional treatment modalities. By targeting key immune checkpoints such as programmed cell death protein 1 (PD-1) and programmed death ligand 1 (PD-L1), these agents have shown remarkable efficacy in patients with advanced gastric cancer, leading to durable responses and improved survival outcomes (12). For example, the CheckMate 649 trial has yielded significant data regarding the efficacy of the PD-1 inhibitor nivolumab in combination with traditional chemotherapy regimens for the management of advanced gastric cancer. The combination therapy of nivolumab with chemotherapy demonstrated notable improvements in overall survival (OS) with a hazard ratio (HR) of 0.71 (98.4% CI 0.59–0.86; *p* < 0.0001) and progression-free survival (PFS) with a HR of 0.68 (98% CI 0.56–0.81; *p* < 0.0001), compared to chemotherapy alone, specifically in patients with a PD-L1 combined positive score (CPS) of five or higher (13). Consequently, regulatory agencies promptly granted approval for this novel therapeutic approach (14). Another example is the KEYNOTE-859 trial, which further solidified the role of PD-1 in combination with chemotherapy for advanced gastric cancer. The study revealed that the median overall survival was 12.9 months and significantly longer in the pembrolizumab group compared to the placebo group among the intention-to-treat population (15). In addition, recent research has demonstrated the significant therapeutic benefits of combining PD-1 and cytotoxic T lymphocyte antigen-4 (CTLA-4) inhibitors in the treatment of advanced gastric cancer (16). PD-1 and CTLA-4 are immune checkpoint proteins that play crucial roles in regulating T cell activation and maintaining immune tolerance (17). By blocking these checkpoint proteins, the immune system’s ability to recognize and attack cancer cells is enhanced. A recent phase three clinical trial (COMPASSION-15) has demonstrated the superior efficacy and safety profile of cadonilimab, a tetravalent anti-PD-1/CTLA-4 bispecific human antibody with enhanced binding affinity for tumor tissue, when used in combination with chemotherapy compared to chemotherapy alone in the treatment of patients with unresectable locally advanced or metastatic gastric cancer. The clinical trial revealed a median PFS of 7.0 months compared to 5.3 months [Hazard Ratio (HR) 0.53, 95% Confidence Interval (CI) 0.44–0.65, 18]. In patients with a PD-L1 CPS ≥ 5, the median OS was 15.3 months versus 10.9 months (HR 0.58, 95% CI 0.41–0.82), with the objective response rate was 65.2% versus 48.9% (18). This result suggests that cadonilimab provides significant benefit in terms of PFS, OS, and objective response rate in patients with a PD-L1 CPS ≥ 5. These results underline the potential clinical efficacy of the treatment regimen under study. However, it is paramount to evaluate the cost-effectiveness of integrating cadonilimab into standard chemotherapy regimens, especially considering the constraints of available resources. Conducting a comprehensive cost-effectiveness analysis can provide critical insights for healthcare practitioners and policymakers, facilitating informed decision-making regarding treatment strategies. Therefore, the objective of this study is to assess the cost-effectiveness of cadonilimab compared to chemotherapy as a first-line treatment for advanced gastric cancer from the perspective of the Chinese healthcare system. This analysis aims to offer valuable information for optimizing treatment approaches and resource allocation in the management of advanced gastric cancer.

## 2 Methods

### 2.1 Study design and model

**Figure 1:**
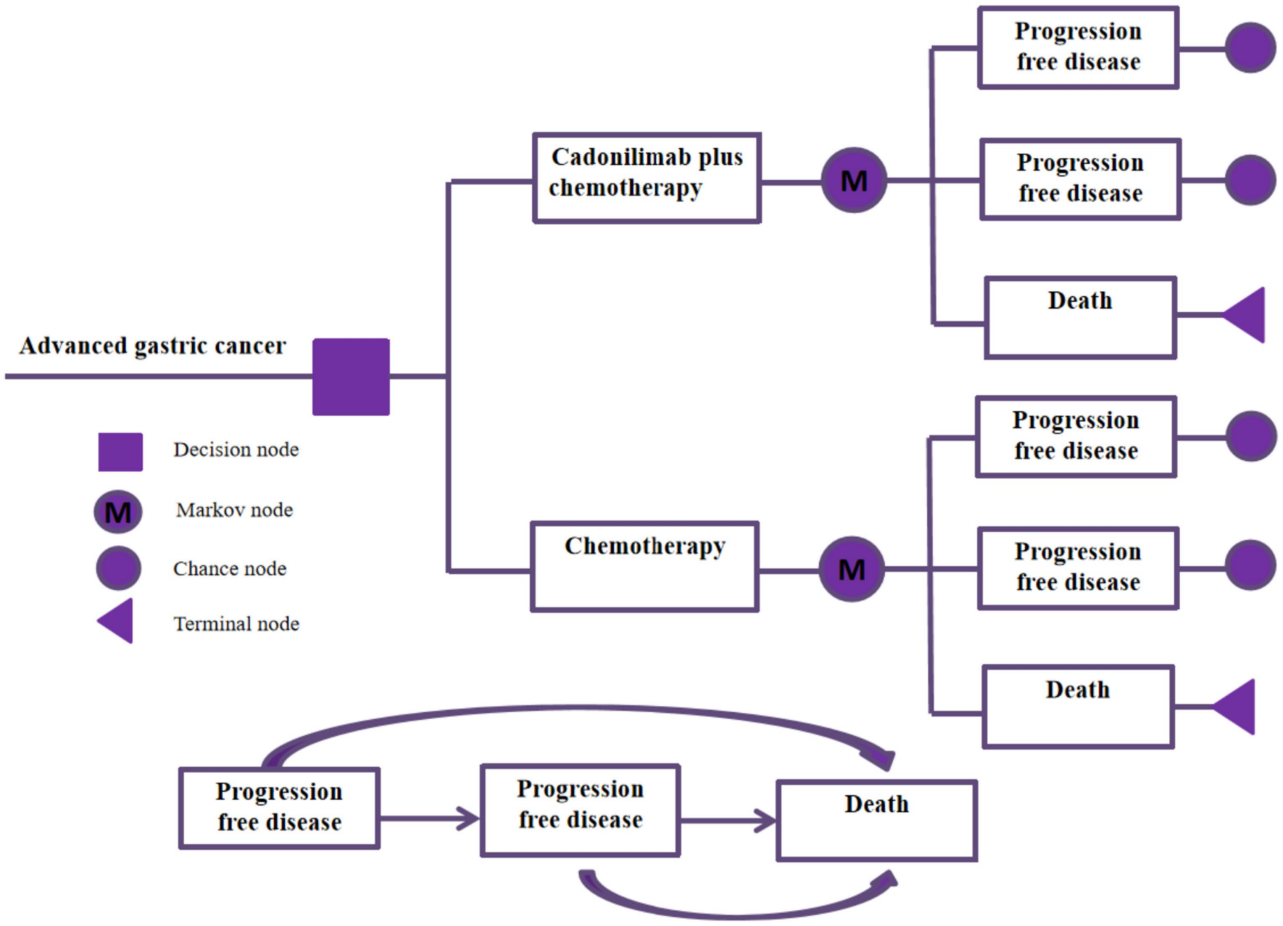
The structure of the model.

Based on the results of the COMPASSION-15 Phase III trial, a partitioned survival model was developed to evaluate the cost-effectiveness of cadonilimab in combination with chemotherapy versus chemotherapy alone for the treatment of advanced gastric cancer. This model incorporates three distinct and mutually exclusive health states: progression-free survival, disease progression, and death. Progression-free survival refers to the period from the initiation of treatment until either tumor progression or patient death. Disease progression is defined by a ≥ 20% increase in the sum of the longest diameters of target lesions or the emergence of new lesions, as outlined by the RECIST (Response Evaluation Criteria in Solid Tumors) guidelines. Disease progression can also be evaluated through a comprehensive assessment of symptoms, clinical signs, and imaging findings, including the development of new lesions or the exacerbation of existing ones. Additionally, death is considered an endpoint in survival analyses, with the time from the commencement of treatment to patient death. Each patient starts in the progression-free survival state at the beginning of the simulation, and transitions between states based on the probabilities of disease progression observed in the clinical trial. During each treatment cycle, it is possible for patients to move to a different state depending on the probability of metastasis observed in the clinical trial. The treatment regimen assigned to each patient corresponds to the health state. Figure 1 represents the structure of the model, which was run using the software TreeAge Pro 2022. In order to capture the long-term cost and benefit outcomes of the treatment strategies, we set the total time horizon of the model to 10 years.

All costs in this study were standardized to US dollars (USD), with any amounts in Renminbi (RMB) converted to USD using the average exchange rate provided by the Bank of China in 2024 (1 USD = RMB 7.12) (19). The willingness-to-pay (WTP) threshold in this study was established at 40,343.68 USD per quality-adjusted life year (QALY). This threshold aligns with the recommendation of the guidelines for pharmacoeconomic evaluation in China, which recommend establishing the WTP threshold at three times the per capita Gross Domestic Product (GDP) (20).

### 2.2 Clinical parameters input

Our study model was conducted on a population that aligns with the COMPASSION-15 Phase III clinical trial cohort. The median age of participants in both the cadonilimab and placebo groups was 64 years. The majority of patients were male, with 78.4 and 77.0% in the cadonilimab and placebo groups, respectively. Additionally, most patients had an Eastern Cooperative Oncology Group (ECOG) performance status score of 1, with percentages of 77.0 and 76.4% in the cadonilimab and placebo groups, respectively. At baseline, 38% of patients in the cadonilimab group and 46% in the placebo group exhibited a PD-L1 CPS of 5 or higher (18).

Patients were randomly assigned at a 1:1 ratio to receive either cadonilimab at a dose of 10 mg/kg every 3 weeks in combination with capecitabine plus oxaliplatin (CAPOX) chemotherapy, or placebo in combination with CAPOX chemotherapy administered every 3 weeks. The CAPOX regimen included oral capecitabine at a dose of 1,000 mg/m^2^ twice daily for 14 days, along with intravenous oxaliplatin at a dose of 130 mg/m^2^ on day 1. Treatment was continued until disease progression or unacceptable toxicity occurred. The median duration of treatment with cadonilimab was 5.62 months (range: 0.5–22.2 months), while the median duration of CAPOX chemotherapy treatment in the cadonilimab group was 4.17 months (range: 0.5–22.2 months) and 4.14 months (range: 0.6–7.1 months) in the control group.

Our cost analysis focused on the economic impact of managing adverse drug reactions (ADRs), particularly grade 3–4 serious ADRs, with an incidence of exceed 10% in both treatment groups. A total of 105 patients (34.4%) in the cadonilimab group and 146 patients (47.9%) in the placebo groupreceived subsequent chemotherapy treatment. As part of the cost analysis, it was assumed that the subsequent regimen for chemotherapy treatment consisted of paclitaxel in combination with cisplatin. Additionally, since the optimal treatment for third-line therapy was not determined through clinical trials, the best supportive treatment was considered as the regimen for third-line therapy. These assumptions will be further explored in sensitivity analyses to ensure the robustness and reliability of the results obtained in this study.

### 2.3 Transfer probability parameters input

The transition probabilities utilized in the partitioned survival model were obtained through the extraction and reconstruction of overall survival (OS) and progression-free survival (PFS) data from the COMPASSION-15 Phase III trial. The OS and PFS survival curves were extracted from the clinical trials using GetData Graph Digitizer (version 2.25) software. The survival curve simulation results were present in Supplementary Figure 1. The internal validation demonstrated that the PFS and OS curves were closely approximated those presented in the clinical trials. Subsequently, the survival curves were refitted using various distribution models (21). We assessed the Akaike Information Criterion (AIC) data and Bayesian Information Criterion (BIC) data across various distribution models in order to determine the model that best fits the data. Lower values of AIC and BIC indicate a better fit of the distribution model to the data (22). Additionally, we conducted a visual inspection of the fitted survival curves for each distribution. Supplementary Table 1 and Supplementary Figure 2 present detailed data of the results.

Finally, the analysis showed that the logical-logistic distribution had the lowest AIC and BIC values, and visual inspection of the 5-year survival rate was consistent with current clinical practice, thus logical-logistic distribution was the most suitable distribution. Subsequently, survival and regression probabilities were directly derived from the reconstructed survival curves over the follow-up period. At the conclusion of the follow-up period, estimates for survival and transfer probabilities were obtained using the log-logistic distribution, expressed as S(t) = 1/(1 + *λ*t*^γ^*) (23). Table 1 presents the values of the parameters γ and λ.

**Table 1:** The parameters of log-logistic distribution.

Subgroup		All randomized patients		PD-L1 CPS ≥ 5 patients	
Parameters		Shape (*γ*)	Scale (λ)	Shape (γ)	Scale (*λ*)
OS curve	Cadonilimab group	1.54	0.013	1.77	0.0087
	Chemotherapy group	1.92	0.010	1.94	0.0096
PFS curve	Cadonilimab group	1.71	0.028	1.80	0.024
	Chemotherapy group	2.29	0.024	2.22	0.028

### 2.4 Costs and utility parameters

The costs analyzed in this study primarily focused on direct medical costs, including costs related to cadonilimab and chemotherapeutic drugs, disposal of adverse drug reactions, subsequent chemotherapy treatment, best supportive care, laboratory examinations, abdominal CT scans, and follow-up. Drug pricing information was obtained from the YAOZHI platform, with the costs used based on the median data of drugs across multiple provinces in China (2025). Chemotherapeutic drug,such as capecitabine and oxaliplatin was administeredat a dose of 1,000 mg/m^2^ twice daily for 14 days and 130 mg/m^2^ on day 1. We assumed the participants had an average weight of 69.6 kg for men and 59.0 kg for women (24), with a body surface area of 1.72 m^2^ to calculate the dose of chemotherapy drugs. Utility values were employed to assess the quality of life in this study, with utility values ranging from 0 to 1. A value of 0 signifies the poorest health state, while a value of 1 represents the best health state. Given that the COMPASSION-15 Phase III trial did not provide specific utility values, values from existing literature were utilized in this analysis. Subsequent sensitivity analyses will be conducted to rigorously evaluate the reliability and robustness of the input parameters utilized in this study. The cost and utility values applied in this research are presented in Table 2.

**Table 2:** The parameters input of the model.

Parameters	Baseline	Range		Distribution	Source
	Value	Minimum	Maximum		
Grade ≥3 adverse drug reactions rate of cadonilimab group[No. (%)]					
Decreased platelet count	87 (28.50)	–	–	Beta	(18)
Decreased neutrophil count	46 (15.10)	–	–	Beta	(18)
Decreased white blood cell count	22 (7.20)	–	–	Beta	(18)
Anemia	31 (10.20)	–	–	Beta	(18)
Grade ≥3 adverse drug reactions rate of chemotherapy group [No. (%)]					
Decreased platelet count	184 (60.50)	–	–	Beta	(18)
Decreased neutrophil count	150 (49.30)	–	–	Beta	(18)
Decreased white blood cell count	143 (47.00)	–	–	Beta	(18)
Anemia	132 (43.40)	–	–	Beta	(18)
Drug costs (USD)					
Cadonilimab (125 mg)	866.01	649.51	1082.51	Gamma	(35)
Capecitabine (500 mg)	0.32	0.24	0.40	Gamma	(35)
Oxaliplatin (50 mg)	7.56	5.67	9.45	Gamma	(35)
Paclitaxel (30 mg)	7.54	5.66	9.43	Gamma	(35)
Cisplatin (10 mg)	1.31	0.98	1.64	Gamma	(35)
Cost of TEAE per cycle(USD)					
Decreased platelet count	1505.92	1129.44	1882.40	Gamma	(36)
Decreased neutrophil count	115.01	86.26	143.76	Gamma	(36)
Decreased white blood cell count	467.86	350.90	584.83	Gamma	(36)
Anemia	468.19	351.14	585.24	Gamma	(36)
Subsequent therapy per cycle (USD)	184.44	138.33	230.55	Gamma	(35)
Best supportive care (USD)	248.00	186.00	310.00	Gamma	(37)
Follow-up cost per cycle (USD)	55.60	41.70	69.50	Gamma	(37)
Laboratory examinations per cycle (USD)	92.50	69.38	115.63	Gamma	(37)
Abdominal CT per cycle (USD)	105.90	79.43	132.38	Gamma	(37)
Utility					
Progression-free disease	0.80	0.60	1.00	Beta	(38)
Progressive disease	0.58	0.44	0.73	Beta	(38)
Decreased platelet count	0.65	0.49	0.81	Beta	(36)
Decreased neutrophil count	0.20	0.15	0.25	Beta	(36)
Decreased white blood cell count	0.20	0.15	0.25	Beta	(36)
Anemia	0.07	0.05	0.09	Beta	(36)
Body surface area (m^2^)	1.72	1.29	2.15	Beta	(37)
Discount rate	0.05	0.04	0.06	Beta	(20)

### 2.5 Sensitivity analysis

In this study, we conducted both one-way sensitivity analysis and probabilistic sensitivity analysis (PSA) to evaluate the robustness of the model outcomes and conclusions. One-way sensitivity analysis involved varying each input parameter by ±25% to observe the resulting impact on the incremental cost-effectiveness ratio (ICER). The result of this analysis was depicted through tornado plots.

PSA was performed using Monte Carlo simulation, with 1,000 iterations to capture the uncertainty in multiple input parameters simultaneously. By randomly sampling from the probability distributions assigned to each input parameter, PSA enabled us to comprehensively evaluate the potential impact of stochasticity on the ICER. The result of the PSA was presented in scatter plots.

## 3 Results

### 3.1 Base case analysis results

In all the randomized patients, the total cost associated with the cadonilimab regimen amounted to 79,474.01 USD, while the chemotherapy regimen incurred a cost of 20,611.40 USD. Treatment with cadonilimab demonstrated an increase of 1.08 QALYs compared to chemotherapy, with an incremental cost of 58,862.61 USD. The incremental cost-effectiveness ratio (ICER) for this group was 54,502.42 USD per QALY. The ICER for this group were found to surpass the WTP threshold of 40,343.68 USD in China. This suggests that the adoption of cadonilimab may not be cost-effective for the all randomized patients.

The sample size for the PD-L1 CPS ≥ 5 subgroup was 256, which was 116 in the cadonilimab group and 140 in the chemotherapy group. In the subgroup with a PD-L1 CPS ≥ 5, the total cost for patients receiving cadonilimab was 58,450.61 USD, whereas those receiving chemotherapy incurred a cost of 22,788.74 USD. Patients treated with cadonilimab experienced an increase in QALYs by 1.33 compared to chemotherapy, with an incremental cost of 35,661.87 USD. The ICER for this subgroup was 26,813.44 USD per QALY. The analysis demonstrates that cadonilimab treatment for the subgroup of patients with a PD-L1 CPS ≥ 5 may be considered cost-effective in China, as the ICER falls below the WTP threshold of 40,343.68 USD. The result of the base case presented in Table 3.

**Table 3:** The base case results.

Subgroup	Group	Cost (USD)	QALYs	Incremental cost (USD)	Incremental QALY	ICER (USD/QALY)
All randomized patients	Cadonilimab group	79,474.01	2.57	58,862.61	1.08	54,502.42
	Chemotherapy group	20,611.40	1.49	–	–	–
PD-L1 CPS ≥ 5 patients	Cadonilimab group	58,450.61	3.05	35,661.87	1.33	26,813.44
	Chemotherapy group	22,788.74	1.72	–	–	–

### 3.2 Sensitivity analysis results

**Figure 2:**
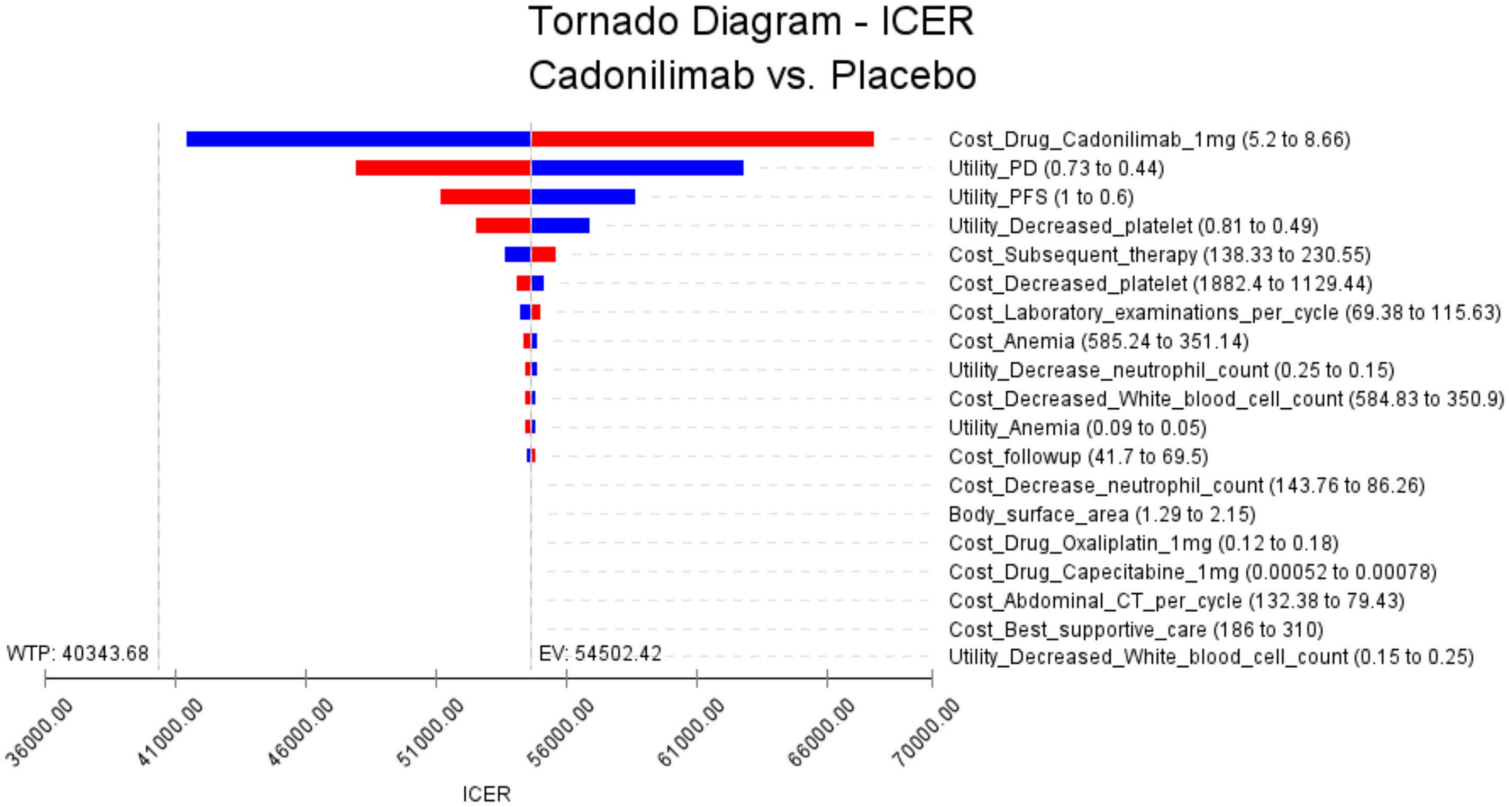
The results of the one-way sensitivity analysis for all randomized populations (The red bars indicate the impact of the parameter on the outcome following an increase in the base value, while the blue bars represent the impact of the parameter on the outcome following a decrease in the base value).

The results of the one-way sensitivity analysis are presented in Figure 2, which illustrates the findings for all randomized patient populations. From these analyses, several key factors were identified that influence the ICER. The most impactful factors were determined to be the cost of cadonilimab, the utility of progressive disease, and the utility of progression-free disease. However, it is noteworthy that even with variations in these key parameters, as well as all other input variables, within a ± 25% range, the resulting ICER values consistently remained above the WTP threshold of 40,343.68 USD. This suggests that changes in these influential factors, as well as other parameters, do not lead to substantial alterations in the ICER outcomes.

**Figure 3:**
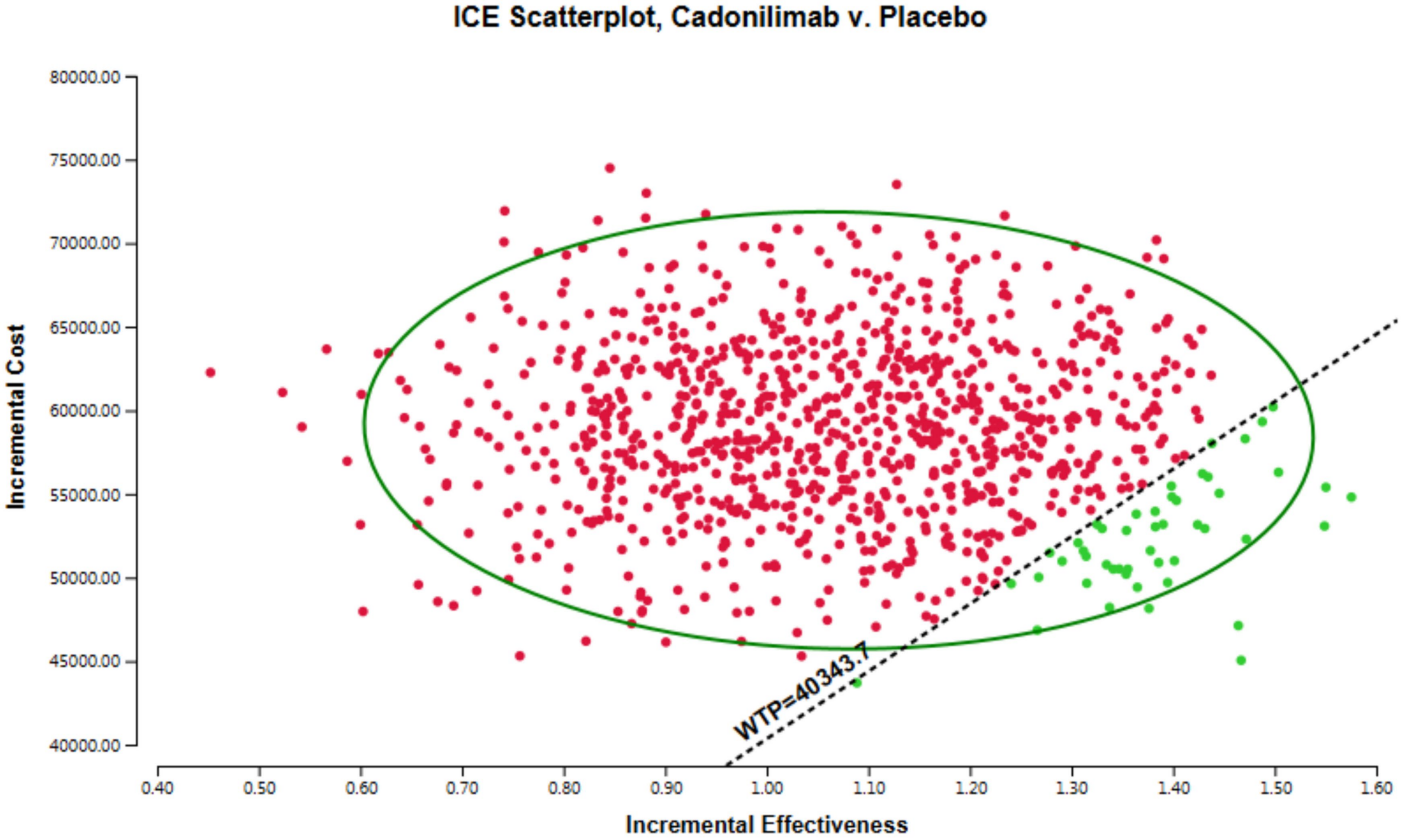
The results of the PSA for all randomized populations.

The results of the PSA are presented in Figure 3, which visually demonstrates the cost-effectiveness advantages of various intervention options in relation to the WTP threshold. Interventions positioned below the linear WTP threshold are considered to have a cost-effectiveness advantage, signaling a more favorable cost-effectiveness ratio. Figure 3 indicates that in the all randomized population, the majority of cadonilimab treatment regimens are situated in the quadrant above the threshold. In addition, the probability of cadonilimab being a cost-effective choice compared to chemotherapy is estimated to be approximately 4.7%. This data suggest that cadonilimab does not currently have a cost-effectiveness advantage over chemotherapy.

### 3.3 Subgroup sensitivity analysis results

**Figure 4:**
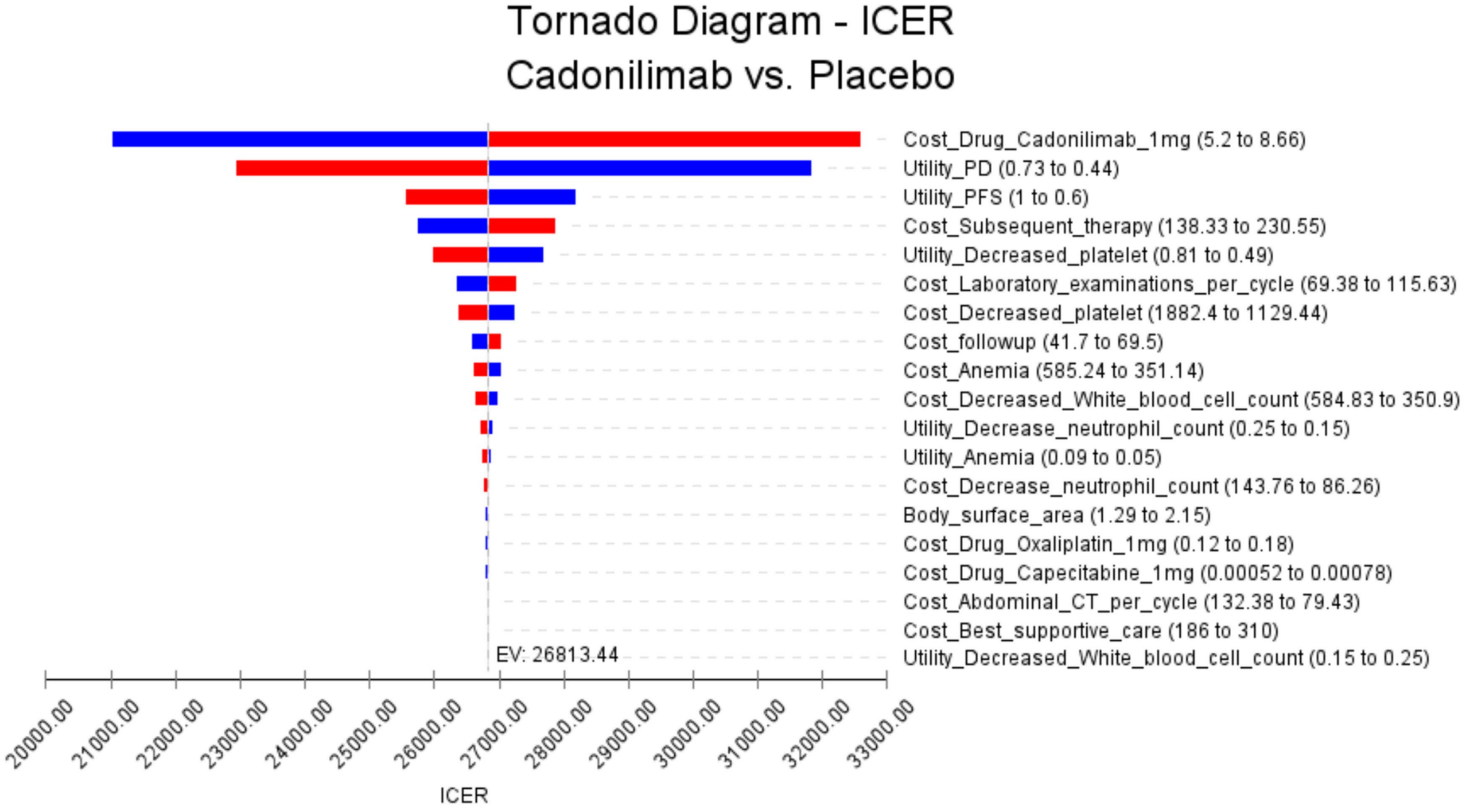
The results of the one-way sensitivity analysis for the PD-L1 CPS ≥ 5 subgroup populations (The red bars indicate the impact of the parameter on the outcome following an increase in the base value, while the blue bars represent the impact of the parameter on the outcome following a decrease in the base value).

The results of the PD-L1 CPS ≥ 5 subgroup one-way sensitivity analysis are presented in Figure 4. Several key parameters were identified as influential in determining the Incremental Cost-Effectiveness Ratio (ICER). Among these, the cost of cadonilimab, the utility of progressive disease, and the utility of progression-free disease were found to have the most significant impact on the ICER. The cost of cadonilimab plays a critical role in influencing the ICER as a major factor. A higher drug cost directly impacts the ICER, making the treatment less economically viable. Furthermore, the utility values of progressive disease and progression-free disease are another impact factors. These values indicate the preference or quality of life associated with different health states. Lower utility values of progressive disease or progression-free disease could result in higher ICERs. This is because a lower quality of life could diminish the cost-effectiveness of the intervention. In addition, compared with all populations, the top three influencing factors of subgroup analysis are the same, but the next influencing factors are slightly different, for example, the cost of Subsequent therapy treatment ranks fourth, and the utility value of decreased platelet count ranks fifth. Notably, even when varying these key parameters and all other input variables within a ± 25% range, the resulting ICER values consistently remained below the WTP threshold of 40,343.68 USD. This suggests that changes in these influential factors, as well as other input variables, do not lead to substantial alterations in the ICER outcomes.

**Figure 5:**
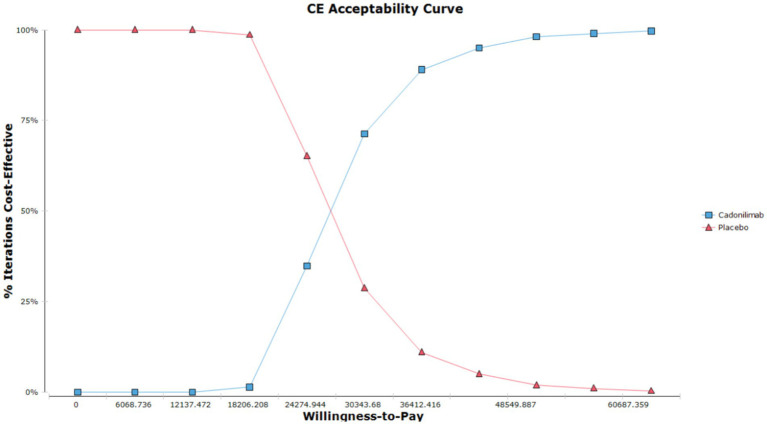
The cost-effectiveness acceptability curve of the PSA for the PD-L1 CPS ≥ 5 subgroup populations.

**Figure 6:**
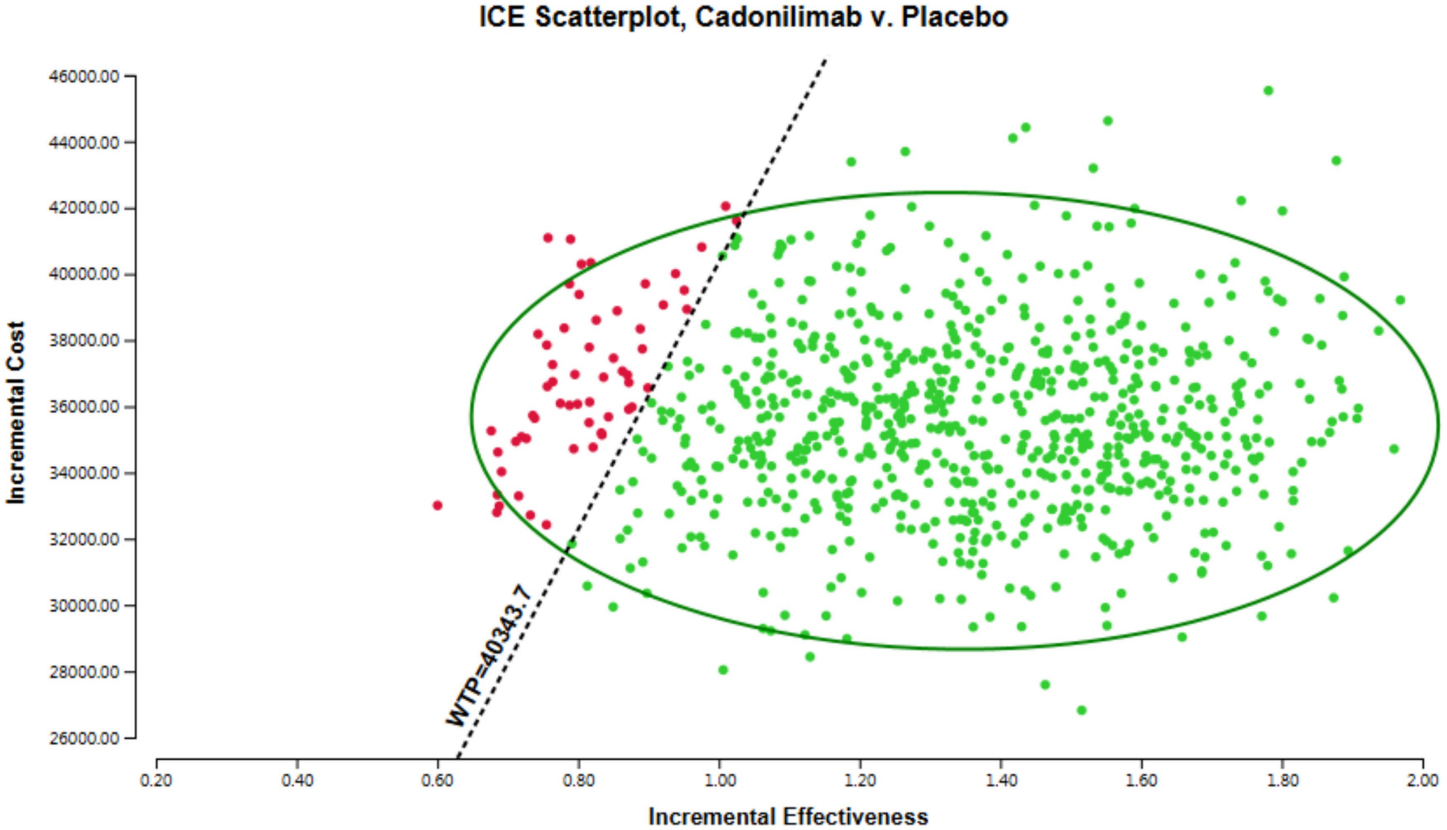
The scatter plot of the PSA for the PD-L1 CPS ≥ 5 subgroup populations.

The results of the probabilistic sensitivity analysis are displayed in Figures 5, 6. Figure 5 presents the cost-effectiveness acceptability curve, illustrating the likelihood of each intervention being cost-effective at varying willingness-to-pay thresholds. Figure 6 shows the probabilistic scatter plot, providing a visual representation of the uncertainty surrounding the cost-effectiveness estimates. The results show that in the PD-L1 CPS ≥ 5 subgroup population, the majority of cadonilimab treatment regimens are situated in the quadrant below the cost-effectiveness threshold. Additionally, the probability of cadonilimab being a cost-effective choice compared to chemotherapy is estimated to be approximately 93.9%.

## 4 Discussion

Gastric cancer remains a significant health burden globally, with a high mortality rate and limited treatment options, particularly in advanced stages of the disease (25). The prognosis for advanced gastric cancer is typically poor, with a 5-year survival below 5%, underscoring the urgent need for improved treatment modalities (26). The emergence of novel therapeutic agents, such as the ICIs cadonilimab, has shown promising results in clinical trials, offering new hope for patients with advanced gastric cancer. The recent COMPASSION-15 phase III trial demonstrated the superior efficacy of cadonilimab in improving PFS and OS compared to chemotherapy. While the clinical benefits of cadonilimab are encouraging, considerations of cost-effectiveness and affordability are paramount in the evaluation of its overall impact on healthcare systems. The rising costs associated with ICIs, including cadonilimab, have raised concerns about the financial implications of incorporating such agents into standard treatment protocols for advanced gastric cancer. Studies have indicated that the costs were observed to increase with advancing stages of the disease, with costs ranging from 2,707 USD for stage I to 4,608 USD for stage IV (27). These findings underscore the importance of evaluating the economic feasibility of novel therapies to ensure optimal resource allocation and sustainability within healthcare systems. Consequently, assessing the cost-effectiveness of treatment options like cadonilimab is essential for informing clinical decision-making and guiding healthcare policies. By balancing the clinical benefits with the economic considerations, stakeholders can optimize the value of healthcare interventions and prioritize interventions that provide the greatest benefit at a reasonable cost.

In this study, the group receiving cadonilimab demonstrated improvements in QALYs compared to the chemotherapy group within both the all randomized populations and the subgroup of patients with PD-L1 CPS ≥ 5. The ICER for the all randomized populations was 54,502.42 USD per QALY gained, while for the PD-L1 CPS ≥ 5 subgroup, the ICER was 26,813.44 USD per QALY gained. The subgroup analysis specifically highlighted that the PD-L1 CPS ≥ 5 group exhibited the lowest ICER among the populations studied, indicating that the cost-effectiveness of cadonilimab treatment in this subgroup is particularly favorable. It is important to note that the ICER for the PD-L1 CPS ≥ 5 subgroup falls below the WTP threshold of 40,343.68 USD. This suggests that treatment with cadonilimab for patients with a PD-L1 CPS ≥ 5 may represent a cost-effective option within in China.

Further sensitivity analysis demonstrates the robustness of our findings. Our study has identified key factors that significantly impact the ICER, with the cost of cadonilimab playing a crucial role in determining the ICER values. Higher drug costs have been shown to result in elevated ICER values, which in turn can affect the economic viability of the intervention. Conducting sensitivity analyses by varying parameters and input variables within a range of ±25% consistently yielded ICER values that exceeded the willingness-to-pay (WTP) threshold of 40,343.68 USD for all randomized patient populations. Interestingly, for the subgroup of patients with PD-L1 CPS ≥ 5, even after adjusting key parameters and all input variables within a ± 25% range, the resulting ICER values consistently remained below the WTP threshold of 40,343.68 USD.

According to the recommendation of the guidelines for pharmacoeconomic evaluation in China, the WTP threshold in this study was established at 3 times the GDP per capita at 40,343.68 USD per QALY. The selection of a WTP threshold is a critical decision that reflects the opportunity cost of health resources and aims to strike a balance between efficiency and equity in resource allocation (28). Firstly, the adoption of a higher threshold, such as three times the GDP per capita, takes into account the budget constraints faced by health systems. Given China’s vast population and disparities in health resource distribution, a 3 × GDP threshold more accurately reflects the marginal health gain per unit of cost, preventing the exclusion of potentially high-impact interventions that could be overlooked with a stricter threshold of 1–1.5 × GDP. Secondly, empirical studies indicate that lower threshold as 1–1.5 × GDP may lead to the exclusion of interventions with significant long-term health benefits, such as cancer treatments or therapies for rare diseases, which could still be cost-effective at the 3 × GDP level (29). Lastly, the adoption of a 3 × GDP threshold helps to strike a balance between equity and efficiency in resource allocation. While a lower threshold as 1–1.5 × GDP prioritizes technical efficiency by maximizing health gains per unit of cost, the 3 × GDP threshold incorporates considerations of equity by allowing for interventions that benefit vulnerable populations, including those in rural or low-income areas (30). In such contexts, the cost per QALY may be higher due to systemic inequalities, and a higher threshold can help address these disparities, aligning with China’s policy objectives of achieving universal health coverage and reducing health inequities.

Although our study did not reveal a cost-effectiveness advantage of cadonilimab over chemotherapy across all randomized populations, it is important to note that cost-effectiveness analysis should not be the sole determining factor in the decision to use cadonilimab. Our recommendation is for policymakers to consider the results of our cost-effectiveness analysis when making decisions about the utilization of cadonilimab, and to explore additional strategies to enhance its economic feasibility and accessibility.

Moreover, our analysis revealed that the subgroup with PD-L1 CPS ≥ 5 exhibited a more favorable cost-effectiveness profile compared to the overall study population. This finding suggests that patients with higher PD-L1 expression levels may derive greater clinical benefit from cadonilimab therapy, making it a potentially more cost-effective treatment option for this specific subgroup. The concept of utilizing biomarkers such as PD-L1 expression levels CPS to guide treatment decisions is well-recognized in the field of oncology (31). By tailoring treatment to individual patients based on their biomarker profile, healthcare providers can optimize therapeutic outcomes while minimizing unnecessary costs and adverse effects. In the case of cadonilimab therapy, our results suggest that targeting patients with PD-L1 CPS ≥ 5 may lead to both clinical and economic benefits, as evidenced by the lower ICER value observed in this subgroup.

There is limited research available on the cost-effectiveness of cadonilimab in the treatment of advanced gastric cancer. However, there is a study have explored the cost-effectiveness of cadonilimab in conjunction with chemotherapy for metastatic cervical cancer. A study conducted by Ding et al. assessed the cost-effectiveness of the addition of cadonilimab to standard chemotherapy compared to chemotherapy alone in the management of metastatic cervical cancer. The results of the study indicated that the combination therapy was not deemed cost-effective in this context, as evidenced by an ICER of 75,944.56 USD per QALY gained (32). In addition, Lang et al. conducted a study evaluating the cost-effectiveness of incorporating tislelizumab in combination with chemotherapy for advanced gastric cancer. Their findings suggested that the inclusion of tislelizumab alongside standard chemotherapy regimens represented a cost-effective therapeutic strategy, with an ICER of 37,768.48 USD per QALY gained (33). This analysis underscores the potential value of integrating sintilimab into the treatment paradigm for advanced gastric cancer, offering valuable insights for clinical decision-making and resource allocation in this patient population. Shu et al. employed a state-transition Markov model to evaluate the cost-effectiveness of nivolumab combined with chemotherapy versus chemotherapy alone as a first-line treatment for advanced gastric cancer, gastroesophageal junction cancer, and esophageal adenocarcinoma. The analysis revealed that nivolumab plus chemotherapy yielded an incremental effectiveness of 0.28 QALYs and an additional cost of 78,626.53 USD, resulting in an ICER of 278,658.71 USD per QALY. This ICER significantly exceeds the WTP threshold of China in 2021, which is set at 31,498.70 USD per QALY (34). Consequently, nivolumab plus chemotherapy does not represent a cost-effective treatment option compared to chemotherapy alone as first-line therapy for patients with advanced gastric cancer, gastroesophageal junction cancer, and esophageal adenocarcinoma in China. Although not a direct head-to-head comparison, current ICER and QALY data suggest that cadonilimab in China showed the potential to be more cost-effective than nivolumab in cost-effectiveness analysis.

There are several limitations to be considered in this study. Firstly, the key survival transition probabilities and clinical data utilized were derived from the COMPASSION-15 clinical trial, which had a limited follow-up period. It is imperative to update this data with findings from long-term follow-up studies to ensure the accuracy and relevance of the results. Secondly, in real-world clinical practice, the selection of second-line therapy for patients post disease progression may vary on an individual basis. Thus, the assumptions and input parameters made in this study could potentially introduce bias between the projected outcomes and actual treatment outcomes. Nonetheless, sensitivity analyses were conducted to assess the impact of these uncertainties, with results indicating that changes in these assumptions did not undermine the robustness of the conclusions drawn. Thirdly, it is important to note that only treatment-related serious adverse reactions of grade three and above were considered in this study, excluding all other adverse reactions. Consequently, there may be a discrepancy between the derived cost and utility values and the actual outcomes observed in clinical practice. Nevertheless, the one-way sensitivity analysis demonstrated that fluctuations in costs associated with grade three or higher adverse events did not significantly alter the ICER. This stability in ICER values underscores the robustness of our conclusions. Finally, for advanced gastric cancer patients, a 10-year time horizon may indeed be insufficient to capture lifetime costs and outcomes, particularly given the emerging long-term survival benefits of immunotherapy. But we also have chemotherapy groups in our model analysis, the standard protocol for advanced or metastatic gastric cancer involved a combination of fluoropyrimidine and platinum-based chemotherapy. Unfortunately, this approach yielded a median overall survival rate of 1 year or less. During the 10-year run of our model, mortality was 67% in the immunotherapy group, but 93% in the chemotherapy group. We also had cycle simulations of 20 years, found that QALY increased with cycle extension in the immunotherapy arm (because there was no complete death in the model, and clinical benefit increased with time) as 3.53QALY and cost of 89025.65 USD, but QALY remained at 1.49 in the chemotherapy arm alone (because there was no complete death in the model, and clinical benefit remained unchanged with cycle extension). This calculated ICER value of 33,536.40 USD/QALY is lower than our result of 54,502.42 USD/QALY, indicating that the cost–benefit advantage for this group of patients widens further with the long-lasting response to immunosuppressant therapy. But limited by survival rates in the chemotherapy arm of the study, we chose a 10-year simulation time. The 10-year model may underestimate the full potential of immunotherapy, as its long-term benefits—including durable responses, immune memory-driven control, and extended survival in specific populations often unfold over more extended periods. Long-term follow-up studies was critical to fully characterizing these advantages.

## 5 Conclusion

The cost-effectiveness analysis has indicated that the incorporation of cadonilimab alongside standard chemotherapy for the initial management of HER-2-negative advanced gastric cancer may not be deemed a cost-effective choice in comparison to chemotherapy alone. However, for a specific subgroup of patients exhibiting PD-L1CPS ≥ 5, the ICER was 26,813.44, signifying that this treatment strategy could potentially be considered cost-effective in China.

## Data Availability

The original contributions presented in the study are included in the article/Supplementary material, further inquiries can be directed to the corresponding author.
