# Supplementary material for: Cost-effectiveness analysis of cadonilimab plus chemotherapy as a first-line treatment option in HER-2-negative advanced gastric cancer
Source: Front Public Health. 2025 Jul 21;13:1644176. doi: 10.3389/fpubh.2025.1644176 (PMC12318942; doi:10.3389/fpubh.2025.1644176)
Supplement: Supplementary file 1 [file Table_1.docx]

**Supplementary Table 1. Comparison of survival models distribution**

|  |  | AIC | | BIC | |
| --- | --- | --- | --- | --- | --- |
| Subgroup | Distribution | Cadonilimab  group | Chemotherap**y** group | Cadonilimab  group | Cadonilimab  group |
| All randomized patients | PFS |  |  |  |  |
|  | Weibull | 1225.148 | 1293.643 | 1232.588 | 1301.084 |
|  | Log-logistic | 1199.409 | 1264.831 | 1206.850 | 1272.272 |
|  | Log-normal | 1205.049 | 1266.537 | 1212.489 | 1273.977 |
|  | Gompertz | 1240.502 | 1326.808 | 1247.943 | 1334.248 |
|  | Exponential | 1241.125 | 1335.670 | 1244.845 | 1339.391 |
|  | Gamma | 1218.019 | 1279.817 | 1225.460 | 1287.258 |
|  |  |  |  |  |  |
|  | OS |  |  |  |  |
|  | Weibull | 1327.582 | 1512.392 | 1335.022 | 1519.832 |
|  | Log-logistic | 1316.691 | 1512.252 | 1324.132 | 1519.692 |
|  | Log-normal | 1319.503 | 1520.487 | 1326.944 | 1527.928 |
|  | Gompertz | 1341.798 | 1526.712 | 1349.239 | 1534.152 |
|  | Exponential | 1349.642 | 1550.913 | 1353.362 | 1554.633 |
|  | Gamma | 1323.464 | 1510.647 | 1330.905 | 1518.088 |
|  |  |  |  |  |  |
| PD-L1 CPS ≥5 patients | PFS |  |  |  |  |
|  | Weibull | 441.052 | 632.028 | 446.559 | 637.911 |
|  | Log-logistic | 430.825 | 631.070 | 436.332 | 636.953 |
|  | Log-normal | 433.061 | 632.673 | 438.568 | 638.556 |
|  | Gompertz | 445.599 | 647.446 | 451.107 | 653.329 |
|  | Exponential | 443.791 | 661.751 | 446.545 | 664.693 |
|  | Gamma | 438.656 | 628.891 | 444.164 | 634.775 |
|  |  |  |  |  |  |
|  | OS |  |  |  |  |
|  | Weibull | 436.470 | 684.275 | 441.977 | 687.158 |
|  | Log-logistic | 431.913 | 681.497 | 437.421 | 687.380 |
|  | Log-normal | 433.830 | 685.974 | 439.337 | 691.858 |
|  | Gompertz | 439.690 | 687.418 | 445.198 | 693.302 |
|  | Exponential | 438.391 | 696.809 | 441.144 | 699.809 |
|  | Gamma | 435.456 | 680.767 | 440.963 | 686.650 |

AIC: Akaike information criterion; BIC: Bayesian Information Criterion; OS: Overall survival; PFS: Progression-free survival;

**
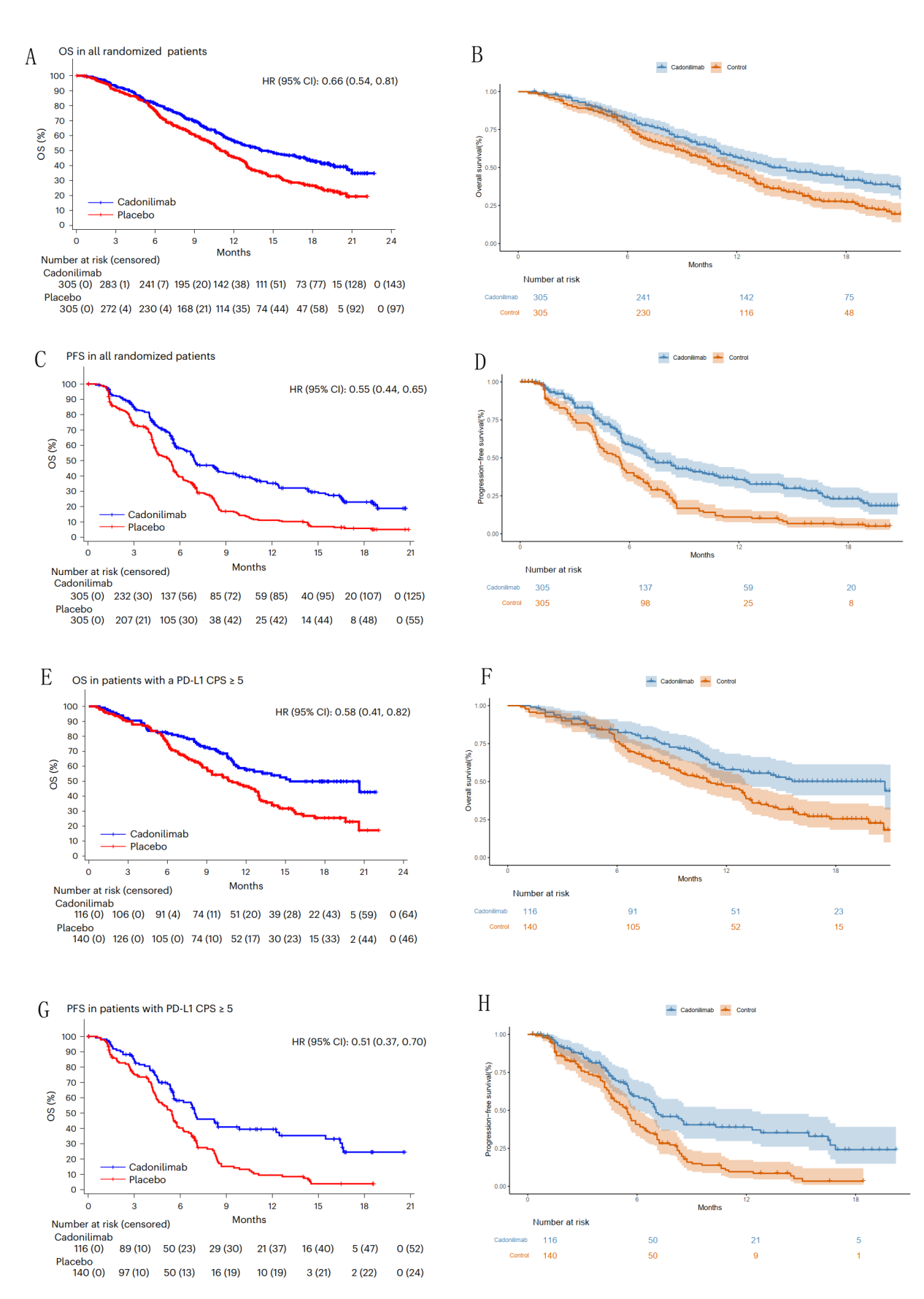
**

**Supplementary Figure 1 The survival curve simulation results .**(A) Kaplan–Meier curve of the overall survival from the COMPASSION-15 Phase III trial for all randomized patients (B) Simulate overall survival curve for all randomized patients (C) Kaplan-Meier curve of progression-free survival from the COMPASSION-15 Phase III trial for all randomized patients (D) Simulate progression-free survival curve for all randomized patients.(E) Kaplan–Meier curve of the overall survival from the COMPASSION-15 Phase III trial for PD-L1 CPS ≥5 patients (F) Simulate overall survival curve for PD-L1 CPS ≥5 patients (G) Kaplan-Meier curve of progression-free survival from the COMPASSION-15 Phase III trial for PD-L1 CPS ≥5 patients (H) Simulate progression-free survival curve for PD-L1 CPS ≥5 patients .


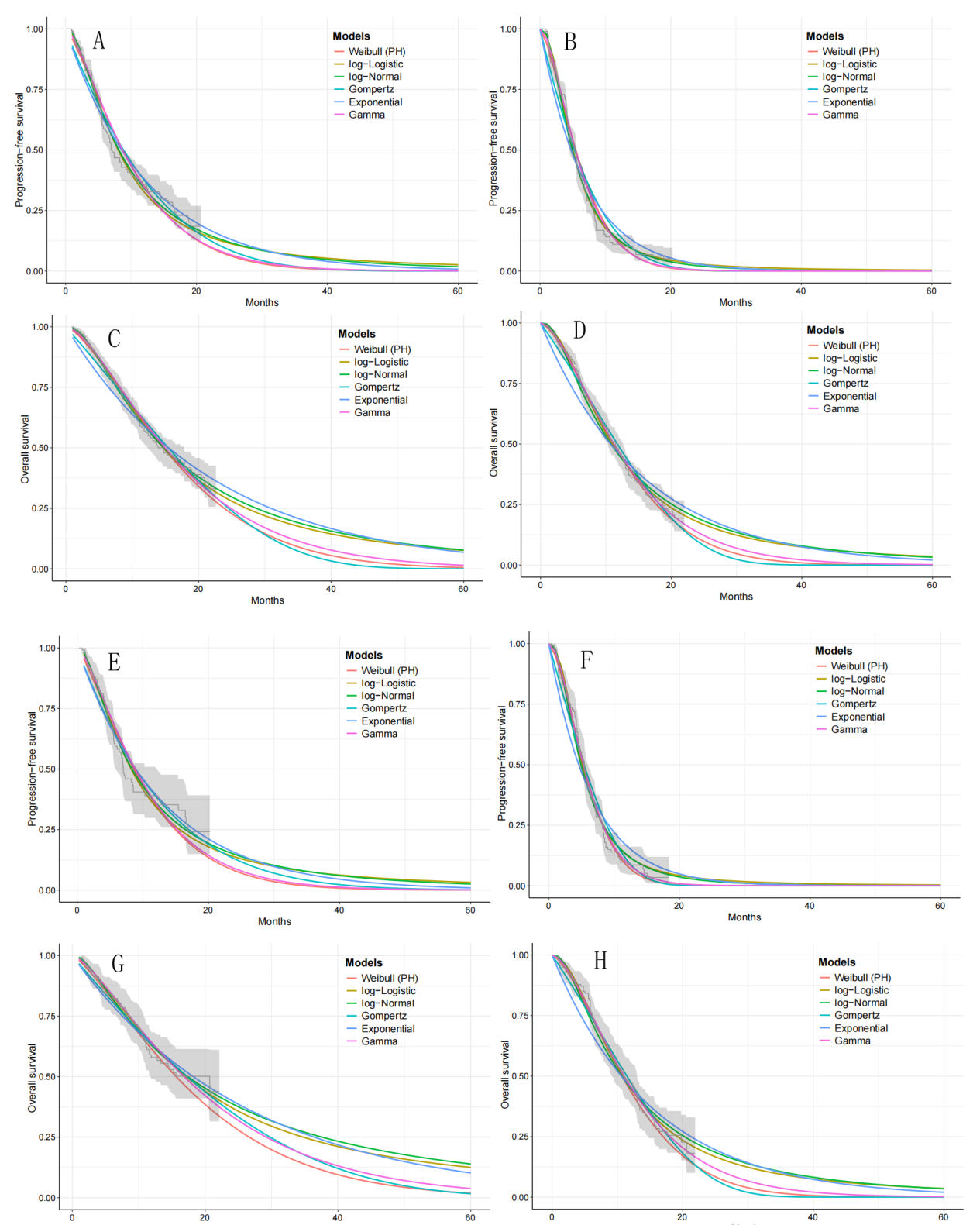


**Supplementary Figure 2** Modes simulation visual survival curve. A:Progression-free survival curve of all randomized patients cadonilimab group;B:Progression-free survival curve of all randomized patients chemotherapy group;C:Overall survival curve of all randomized patients cadonilimab group;D: Overall survival curve of all randomized patients chemotherapy group;E:Progression-free survival curve of PD-L1 CPS ≥5 patients cadonilimab group;F:Progression-free survival curve of PD-L1 CPS ≥5 patients chemotherapy group;G:Overall survival curve of PD-L1 CPS ≥5 patients cadonilimab group;H: Overall survival curve of PD-L1 CPS ≥5 patients chemotherapy group;
